# A Case of Diabetic Ketoacidosis With Persistent Alkalosis

**DOI:** 10.7759/cureus.80470

**Published:** 2025-03-12

**Authors:** Alison Southern, Logan O'Keefe

**Affiliations:** 1 Emergency Department, Aultman Hospital, Canton, USA

**Keywords:** critical emergency medicine, diabetic gastroparesis (dg), diabetic ketoacidosis (dka), diabetic ketoalkalosis, nonadherence, type 1 diabetes mellitus

## Abstract

Diabetic ketoacidosis (DKA) is an emergent complication of diabetes. Patients with DKA typically have an arterial pH of 7.30 or lower, caused by the overproduction of β-hydroxybutyric and acetoacetic acids. We present a case of a 24-year-old male with a history of uncontrolled type 1 diabetes mellitus, noncompliance, and gastroparesis who presented to an emergency department with nausea, nonbilious vomiting, and abdominal pain. The physical exam was remarkable for dry mucous membranes, tachycardia, and mild diffuse abdominal tenderness. Initial bloodwork indicated hyperglycemia, increased levels of beta-hydroxybutyric acid, and an elevated anion gap; however, the arterial pH was alkalotic. This led to the suspicion of a combined acid-base disturbance, where profuse vomiting resulted in alkalosis, masking the expected acidosis. The patient was started on intravenous fluids and an insulin drip and was subsequently admitted to the medical intensive care unit. He left the hospital against medical advice two days later. This case is notable because the patient met all the criteria for DKA yet exhibited an alkalotic pH. It highlights the necessity of using the entire clinical picture and not solely relying on laboratory values when diagnosing and treating disease.

## Introduction

Diabetic ketoacidosis (DKA) is a life-threatening complication of diabetes [1]. The expected laboratory values in DKA include an arterial pH of 7.30 or lower and acidosis, which is caused by the overproduction of β-hydroxybutyric and acetoacetic acids. This case challenges the conventional understanding of DKA with a presenting alkalosis rather than acidosis. Combined acid-base disorders may occur in DKA, some resulting in alkalosis [2]. Maintaining a high index of suspicion for DKA is crucial even when the laboratory values do not reveal acidosis.

## Case presentation

We present a case of a 24-year-old male who presented to an emergency department with nausea, nonbilious vomiting, and abdominal pain that began two days prior to his visit. He has a past medical history of uncontrolled type 1 diabetes mellitus, gastroparesis, and marijuana use. His medical history was complicated by a pattern of nonadherence to medical advice, treatment plans, and follow-up care. The patient has had multiple hospital admissions, many of which ended prematurely as he left against medical advice. Despite extensive discussions with the patient and his family, efforts to improve his adherence to medical recommendations have been unsuccessful.

On examination, the patient appeared listless and had shallow respirations. Vital signs included a blood pressure of 137/65 mmHg, heart rate of 116 beats per minute, respiratory rate of 22 breaths per minute, temperature of 37.3°C, and pulse oximetry of 94% on room air. The physical examination showed dry mucous membranes, tachycardia, and mild abdominal tenderness without rebound or guarding. He received 2 L of intravenous normal saline and antiemetics. The initial bloodwork showed a glucose level of 295 mg/dL, an anion gap of 24 mEq/L, and a ß-hydroxybutyrate level greater than 90 mg/dL. Serum potassium was 3.0 mEq/L. Despite the expected acidosis, his venous blood gas analysis was significant for a pH of 7.657. His laboratory values are shown in Table 1.

**Table 1:** Laboratory values of the patient

Test	Patient Value	Reference Range
Random blood sugar	295 mg/dL	70–110 mg/dL
Venous pH	7.657	7.38–7.46
ß-hydroxybutyrate	> 90 mg/dL	0.20–2.81 mg/dL
Sodium	142 mEq/L	136–145 mEq/L
Potassium	3.0 mEq/L	3.5–5.0 mEq/L
Chloride	88 mEq/L	98–110 mEq/L
CO_2_	30 mEq/L	22–32 mEq/L
Creatinine	5.19 mg/dL	0.60–1.40 mg/dL
Anion gap	24 mEq/L	4.0–15.0 mEq/L

The patient's history of present illness, physical examination, and laboratory values suggest DKA. The elevated anion gap and β-hydroxybutyrate support DKA, but the unexpectedly high pH led to the diagnosis of a mixed acid-base disorder.

He was started on an insulin drip with supplemental potassium to treat DKA. The patient was admitted to the medical intensive care unit for ongoing management of DKA. He left the hospital against medical advice two days later.

This hospitalization marked his 12th admission in the past six months. His venous pH levels have ranged from 7.377 to 7.737, and his arterial pH levels have ranged from 7.434 to 7.694.

## Discussion

In DKA, the amount of effective insulin is reduced while concentrations of counterregulatory hormones are increased. These hormones contribute to hyperglycemia and ketosis through heightened gluconeogenesis, accelerated glycogenolysis, and impaired glucose utilization by peripheral tissues [3]. Additionally, free fatty acids are released into circulation from adipose tissue, which the liver then oxidizes into ketone bodies, including β-hydroxybutyrate and acetoacetate [1]. This process ultimately leads to ketonemia and metabolic acidosis [4].

Comparative laboratory values between this patient and a patient with typical DKA are detailed in Table 2. Previous studies have indicated that combined acid-base disorders in DKA can occur in as many as 47.5% of cases, with alkalosis being the most prevalent [2]. Ketoalkalosis has been referred to as "masked DKA" or "alkaline ketoacidosis" [5,6]. It is believed that profuse vomiting, a common symptom of DKA, causes fluid contraction alkalosis and depletion of chloride ions, which may indirectly stimulate bicarbonate resorption [2]. Excessive vomiting can also lead to renal potassium secretion, further contributing to bicarbonate resorption [2]. Given the intractable nausea and vomiting experienced by the patient, there may be volume depletion. This volume depletion can result in hyperaldosteronism, which induces an exchange of sodium ions for potassium or hydrogen ions [2]. All of these metabolic changes ultimately lead to a state of alkalemia rather than acidemia [2]. It has also been noted that patients with a history of autonomic neuropathy, such as gastroparesis, may present with recurrent vomiting that can precipitate alkalemia, as seen in this case [2]. Watanabe et al. documented the cases of two young women with type 1 diabetes mellitus who presented with alkalosis/alkalemia [5]. Another report by Kumar et al. supports these findings [2]. While these cases have been described in endocrinology journals, they are rarely encountered in the emergency medicine literature.

**Table 2:** Patient's laboratory values vs. laboratory values of a patient with typical DKA DKA: diabetic ketoacidosis

Test	Patient Value	Example of Typical DKA	Reference Range
Random blood sugar	295 mg/dL	> 250 mg/dL	70–110 mg/dL
Venous pH	7.657	< 7.30	7.38–7.46
B-hydroxybutyrate	> 90 mg/dL	High	0.20–2.81 mg/dL
Potassium	3.0 mEq/L	High or low	3.5–5.0 mEq/L
CO_2_	30 mEq/L	< 15 mEq/L	22–32 mEq/L
Anion gap	24 mEq/L	High	4.0–15.0 mEq/L

## Conclusions

This case demonstrates that alkalosis does not exclude the possibility of DKA. Maintaining a high level of clinical suspicion for DKA is essential, even when a laboratory value deviates from the expected range. In our patient, the treatment approach did not differ from that typically used for DKA, and treatment modifications were not necessary. Patients should receive aggressive fluid hydration, intravenous insulin, and electrolyte replacement. DKA results in significant morbidity and mortality. Future studies should evaluate how often alkalosis-masked DKA occurs.

Promptly recognizing and treating DKA is crucial; we hope this unusual case serves as a valuable resource for emergency practitioners.
